# Identification and Comparison of Aberrant Key Regulatory Networks in Breast, Colon, Liver, Lung, and Stomach Cancers through Methylome Database Analysis

**DOI:** 10.1371/journal.pone.0097818

**Published:** 2014-05-19

**Authors:** Byungtak Kim, Seongeun Kang, Gookjoo Jeong, Sung-Bin Park, Sun Jung Kim

**Affiliations:** Department of Life Science, Dongguk University-Seoul, Seoul, Korea; University of North Carolina School of Medicine, United States of America

## Abstract

Aberrant methylation of specific CpG sites at the promoter is widely responsible for genesis and development of various cancer types. Even though the microarray-based methylome analyzing techniques have contributed to the elucidation of the methylation change at the genome-wide level, the identification of key methylation markers or top regulatory networks appearing common in highly incident cancers through comparison analysis is still limited. In this study, we in silico performed the genome-wide methylation analysis on each 10 sets of normal and cancer pairs of five tissues: breast, colon, liver, lung, and stomach. The methylation array covers 27,578 CpG sites, corresponding to 14,495 genes, and significantly hypermethylated or hypomethylated genes in the cancer were collected (FDR adjusted p-value <0.05; methylation difference >0.3). Analysis of the dataset confirmed the methylation of previously known methylation markers and further identified novel methylation markers, such as GPX2, CLDN15, and KL. Cluster analysis using the methylome dataset resulted in a diagram with a bipartite mode distinguishing cancer cells from normal cells regardless of tissue types. The analysis further revealed that breast cancer was closest with lung cancer, whereas it was farthest from colon cancer. Pathway analysis identified that either the “cancer” related network or the “cancer” related bio-function appeared as the highest confidence in all the five cancers, whereas each cancer type represents its tissue-specific gene sets. Our results contribute toward understanding the essential abnormal epigenetic pathways involved in carcinogenesis. Further, the novel methylation markers could be applied to establish markers for cancer prognosis.

## Introduction

Carcinogenesis is a complicated cellular process starting from genesis of a few cancer cells and developing into malignancy through cellular proliferation [1], [2]. Depending on the specific tissue and cancer stage, different sets of genes act on the cancerous cells in different modes [3]. For example, BRCA1 is a well-known tumor suppressor gene implicated in the predisposition of early-onset breast and ovarian cancer [4]. On the contrary, loss of RB is well documented in many human tumor types [5].

In the course of cancer development, tumor suppressors as well as proto-oncogenes undergo genetic and epigenetic changes [6]–[9]. Promoter methylation is an epigenetic regulation, and its abnormal changes in cancer-related genes have been known to have pivotal roles during carcinogenesis. So far, numerous genes have been identified as epigenetic markers that can predict occurrence of specific cancers [10]–[13]. BRCA1 is a tumor suppressor, and it is hypermethylated in a limited number of cancer types [14], [15]. Meanwhile, another tumor suppressor, PTEN, is hypermethylated in a wide range of tumor types [16]–[18]. The methylation change of tumor-related genes may appear at a specific stage or sub-tissue. For example, methylation of RASSF1 was observed with gradual increase in association with increasing tumor size and advancement of the stage of breast cancer [19]. In another study, ADAM33 was shown to be silenced by the promoter methylation with hypermethylation in invasive lobular carcinoma compared to invasive ductal carcinoma [20].

These facts imply that specific set of genes are differentially methylated depending on the specific cancer type. Studies chasing methylation change have been extensively carried out in each type of cancer tissue. However, comprehensive approaches wherein the methylation profiles are compared to thus reveal the commonalities in various cancers or cancer-specific epigenetic pathways have been limited. Accumulation of methylation markers in cancer prompts us to establish a systemic approach that is able to predict cancer-related genes and pathways with abnormal methylation. Current high throughput techniques have enabled us to identify genes that show differential methylation in cancer tissues, and genome-wide methylome data are available in public databases such as the Gene Expression Omnibus (GEO) [21]. In this study, by utilizing the GEO methylome databases, we have aimed to elucidate the molecular pathway in which genes are highly dysregulated by promoter methylation and to compare them in the five major cancers: breast, colon, liver, lung, and stomach. To do this, we first analyzed in silico the Illumina methylation bead chip data from five cancers. After collecting data for ten pairs of normal and cancer tissues from each cancer type we compared the interrelationship of the five cancers based on methylomic change. Further, the Ingenuity pathway analysis was carried out to elucidate the top pathway that is eligible to be distorted by methylation.

## Materials and Methods

### Cell culture

MCF10A (normal breast), MCF7 (breast cancer), and HEK293 were purchased from the American Type Culture Collection (ATCC; Manassas, VA). HEL 299 (normal lung) and A549 (lung cancer), and AGS (stomach cancer), THLE-3 (normal liver) and Hep3B (liver cancer), CCD-18Co (normal colon) and HT-29 (colon cancer) were purchased from the Korean Culture Type Collection (KCTC; Korea). MCF10A was grown in MEBM supplemented with MEGM Single Quots and cholera toxin (Lonza, Basel, Switzerland). HEK293, Hep3B, THLE-3, and CCD-18Co were grown in Dulbecco's modified Eagle's medium (DMEM) supplemented with 10% fetal bovine serum (FBS). MCF7, A549, AGS, and HT-29 were cultured in RPMI-1640 supplemented with 10% FBS.

### Microarray database analysis for genome-wide methylation and expression assay

The Illumina Infinium Methylation Chip data for breast, colon, liver, lung, and stomach cancer were obtained from the GEO database (http://www.ncbi.nlm.nih.gov/geo/). Microarray data for ten sets of normal tissue and its matching cancer tissue for each cancer type (fifty sets in total) were collected. The accession numbers of the adopted databases are indicated in Table S1. The Illumina Infinium Methylation Assay detects genome-wide methylation covering 27,578 CpG sites in 14,495 genes. A methylation index (β) was outputted for each site, which is a continuous variable ranging between 0 (no methylation) and 1 (100% methylation), representing the ratio of the intensity of the methylated-probe signal to the total locus signal intensity. These values were then used to calculate a ratio of relative methylation between normal and tumor tissue, with higher values corresponding to greater levels of methylation in tumor tissue relative to normal tissue.

In silico expression analysis for selected genes was carried out on the Oncomine database platform (https://www.oncomine.org). To do this, datasets from the corresponding cancer type were collected for each gene, and expression was examined with a threshold satisfying p<0.01.

### Pathway analysis

To identify pathways displaying methylation-specific altered expression patterns with potential roles in the five cancer types, functional categorization and pathway constructions were conducted using the Ingenuity Pathway Analysis (IPA) software tool produced by Ingenuity Systems as previously described [22]. A repertoire of genes from each cancer type, which was selected as differentially methylated genes was submitted to the IPA. The p-value for individual network was obtained by comparing the likelihood of obtaining the same number of transcripts or greater in a random gene set as are actually present in the input file (i.e., the set of genes differentially expressed in the normal tissue and cancer tissue), using Fischer's exact test based on the hypergeometric distribution. The highest confidence functional network was designated as the top network.

### Real-time RT-PCR

Isolation of Total RNA from cell culture and reverse transcription were conducted as described previously [22]. Expression levels of selected genes were measured by real-time quantitative RT-PCR analysis. At least triplicate reactions were performed for each sample using a Kapa SYBR Fast qPCR Kit (Kapa Biosystems, Woburn, MA) with gene-specific primers (Table S2) on an ABI 7300 instrument (Applied Biosystems). RNA quantity was normalized to GAPDH content, and gene expression was quantified according to the 2^−ΔCt^ method.

### Statistical analysis

A hierarchical clustering dendrogram was generated by Gene Cluster 3.0 (http://bonsai.hgc.jp/~mdehoon/software/cluster/software.htm) to determine the relationship between the genome-wide methylation trend and cancer sample. In the analysis of microarray database, batch effects resulting from the use of different arrays at different tissues and time points were removed using ComBat [23]. Observations with adjusted p-values equal to or greater than 0.05 were removed and thus precluded from further analysis. Following adjustment, the remaining genes were defined as differentially methylated if [β (cancer) – β (normal)] >0.3. A student's t-test was used to detect differences in the mean levels of methylation for selected genes, as well as for the expression level between the control and cancer cells. Statistical analyses were conducted using SPSS for Windows, release 17.0 (SPSS Inc., Chicago, IL). A *p*<0.05 was considered to be statistically significant.

## Results

### In silico methylome analysis identifies tissue-specific methylation profile in breast, colon, liver, lung, and stomach cancer

To identify and compare essential pathways that are deregulated by abnormal methylation of CpGs at the promoter in the major five cancers, breast, colon, liver, lung, and stomach, a repertoire of genome-wide methylation databases was first collected from the GEO database (http://www.ncbi.nlm.nih.gov/geo/) that harbors the Illumina methylation array data covering 14,495 genes and 27,578 CpG sites. Datasets for ten pairs of cancer tissues and the corresponding normal tissues were collected for each cancer type except for breast cancer, in which case the data consisted of only one normal tissue sample and ten cancer tissue samples due to the lack of normal/cancer pairs.

**Figure 1:**
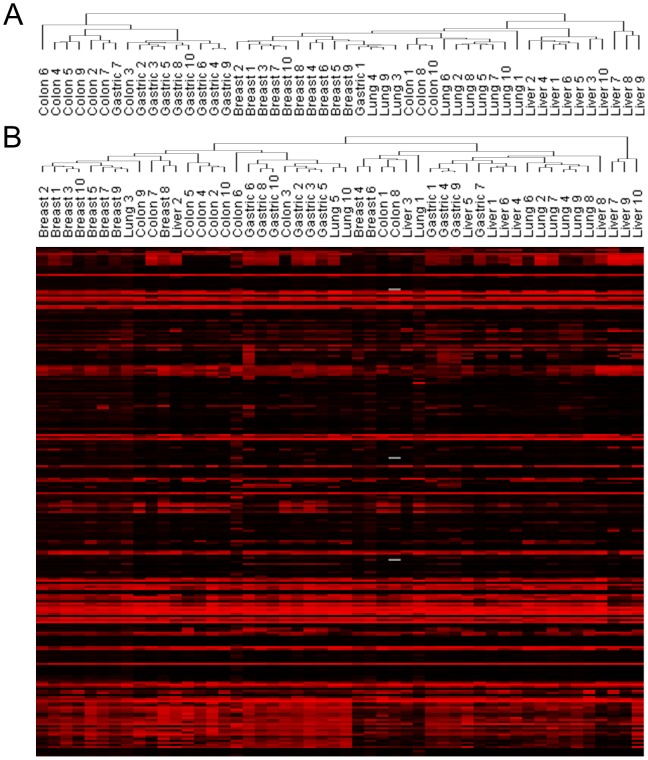
Cluster and heat map analysis with genes of altered methylation for five cancers. A. Cluster analysis was carried out using a methylation index (β-value) taken from Illumina Human Methylation 27 Bead Chip database registered at the GEO. Hierarchical clustering with the correlation distance was shown for 50 tumor tissues from the breast, colon, liver, lung, and stomach cancer. B. Hierarchical cluster analysis was conducted for 90 genes that were related with “cancer” from the five cancer types. The bottom panel is a heat map analysis wherein the rows and columns represent genes and cancer cell lines, respectively.

To get insight into the relationship between the overall methylation change and each of the five cancer types, and further into the relative distance of the cancers, a cluster analysis was carried out. In the cluster, the normal and cancer tissues were generally separated in a bipartite mode, indicating that genome-wide methylation change is a key event distinguishing cancer cells from normal cells regardless of tissue types (Fig. S1A). Moreover, each cancer type was generally grouped together, thus explaining the tissue-specific methylomic profile in both normal and cancer cells (Fig. 1A). A few cancer tissues were grouped nearby other tissues. For example, three colon cancer tissues were separated from the others and grouped together with lung cancers, showing hypermethylation in the VHL and PRDM15 (Fig. S1B). VHL encodes von Hippel-Lindau tumor suppressor and was shown to be hypermethylated in lung cancer [24]. The two subgroups of lung cancer tissues separated by the three colon cancers showed different cancer stages, with one subgroup including only stage I and with the other subgroup including also stage II and III.

Interestingly, we found that breast cancer was closest with lung cancer whereas it was farthest from colon cancer. Next, we selected 90 key cancer-related genes and carried out the cluster analysis for cancer tissues to examine whether they could possibly represent the whole methylation status of cancer tissue. The results indicated a high ratio of overlapping between the two cluster analyses with the whole genome and the cancer-related genes (Fig. 1B).

### Aberrantly methylated genes are involved in cancer-related pathways

To construct the highest functional network in the five cancers, a collection of genes with differential changes of methylation are compared to the corresponding normal tissue, which was selected after filtering off the statistically insignificant genes (*p*>0.05). In each cancer, the filtered genes were compared with another tissue, and genes appearing common in the compared tissues were selected. This comparison step was repeated using the ten normal–cancer pairs until less than 500 genes were filtered. At the end of filtration 3–4 pairs were included to construct the network.

**Figure 2:**
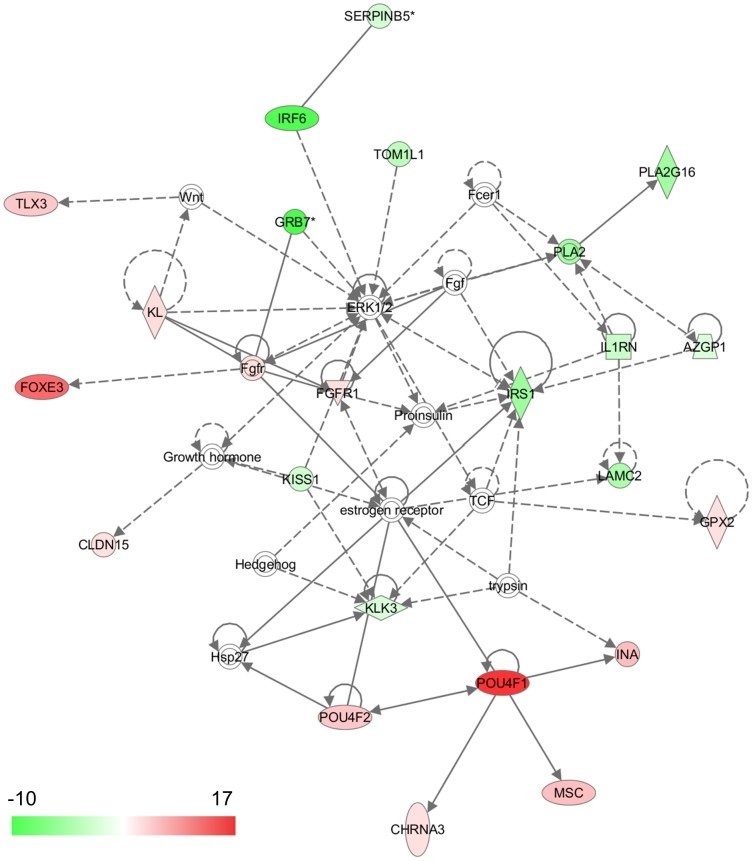
Highest confidence network of genes displaying altered methylation levels in breast cancer. In the network, hypermethylated genes in cancer are colored in red, whereas the hypomethylated genes are shown in green. The intensity of the color reflects the magnitude of methylation change. According to IPA, the network is relevant to “cellular movement, cellular development, and cancer.” The highest confidence network for the other four cancers is added in Fig. S2. Each interaction is supported by at least one literature reference, with solid lines representing direct interactions, and dashed lines representing indirect interactions.

**Figure 3:**
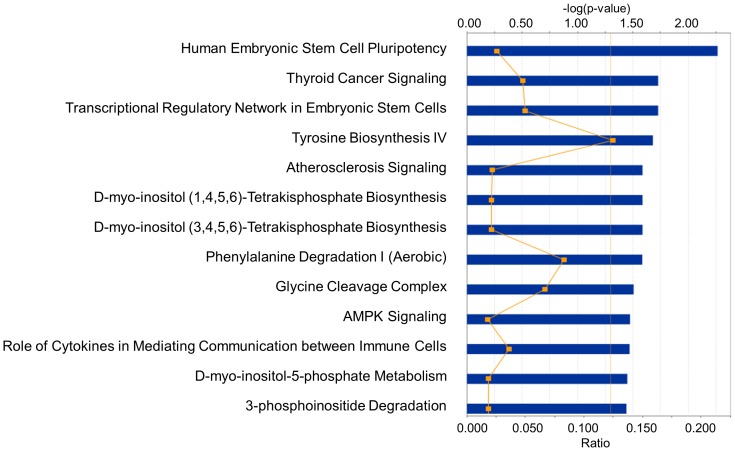
Pathways most strongly associated with the significantly altered genes in the five cancers. Top functional categories are given for breast cancer. The Ingenuity software assigns a *p*-value based on the likelihood of obtaining the observed number of category or pathway-related molecules in a given data set by chance alone. The threshold line denotes the *p* = 0.05 level. The line graph represents the ratio of affected genes to the total number of genes in a pathway. The top functional category for the other four cancers is added in Fig. S3.

All of the sites that fit our significance criteria for differential methylation were examined for functional analysis using the IPA software tool. Of note, the “cancer” related network or bio-function appeared as the highest confidence among all the five cancers (Fig. 2, Fig. 3, Fig. S2, and Fig. S3).

In breast cancer, ER-regulated genes including FGFR1 and IRS1, and ERK1-regulating genes including IRF6 and TOM1L1, were aberrantly methylated. In addition, centered in the network is POU4F1, which interacts with ER and regulates INA, MSC, and CHRNA3.

In lung cancer, two HOX genes, HOXA7 and HOXB4, were highly methylated. Meanwhile, FOSL1 and THRB were hypomethylated, taking centers in the network and appearing to function as the master regulator of a network of several gene transcripts potentially relevant to lung tumor development and progression. In colon cancer, it is remarkable that AR is hypermethylated and acts as an interaction hub in the top network. Activation of AR in colon tumor is known to be a prosurvival signal, and it blocks migration [25]. PKA and ADC were also hypermethylated, which interacted with AR. In gastric cancer, VIM, which has been known to be a methylation marker, was also hypermethylated, and it centered in the network interaction with other proteins, such as PDGF complex and TWIST1. In liver cancer, various kinds of GPCR, including ADRB1 and DRD1PLC and their downstream element PLC, were abnormally methylated.

### Abnormally methylated genes are deregulated in cancer

**Figure 4:**
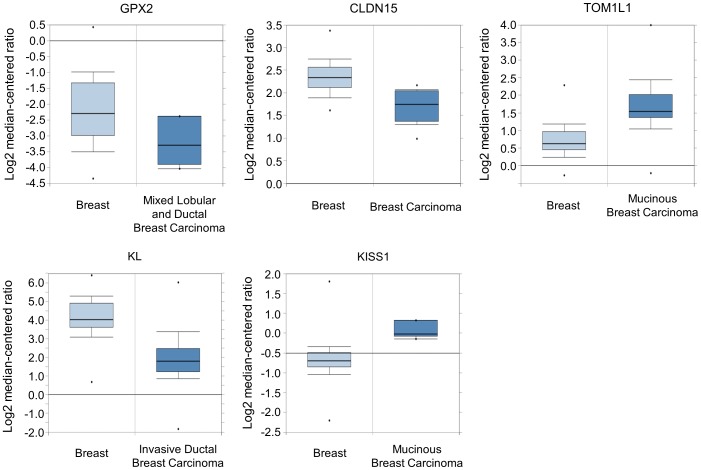
In silico expression profiles of selected genes with altered methylation in breast cancer. Five genes that have shown methylation change in breast cancer were selected, and their expression was examined in silico in the expression dataset and represented as a box plot. There appeared a coincidence that hypermethylated genes showed downregulation, or that hypomethylated genes showed upregulation in cancer. Data for the other four cancers is added in Fig. S4.

**Figure 5:**
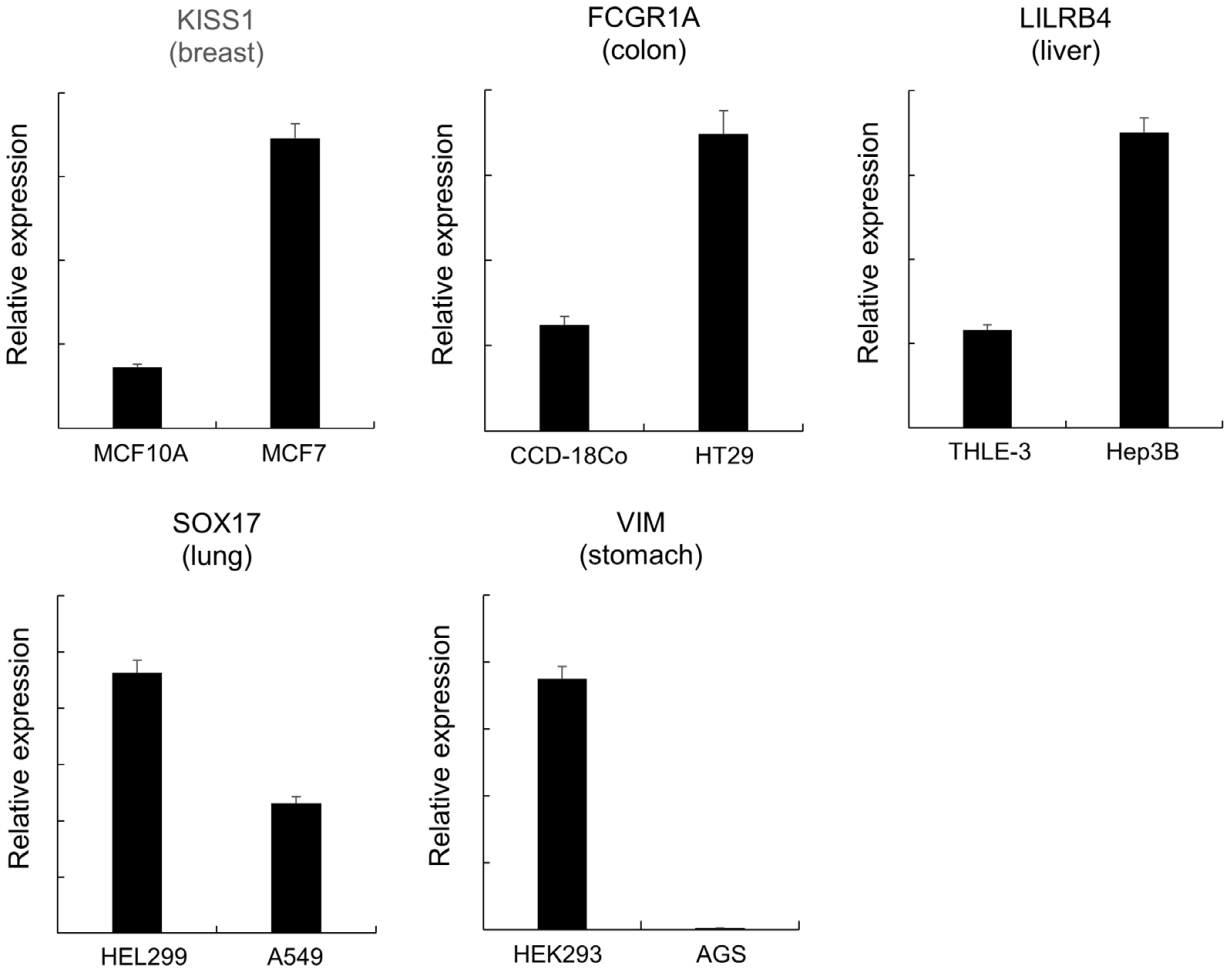
RT-PCR analysis of selected genes that showed altered methylation in the five cancer types. The expression level of selected genes was examined using real-time RT-PCR in a normal and a cancer cell line for the five cancer types. In case of stomach, HEK293 was used as the normal cell line. There appeared a coincidence that hypermethylated genes showed downregulation, or that hypomethylated genes showed upregulation in cancer. Each sample was examined in triplicate, and the average expression levels are presented with standard errors.

To examine whether the abnormally methylated genes induce deregulation of gene expression, in silico dataset analysis as well as real-time RT-PCR was conducted. Twenty-two genes, five from each cancer (two in case of liver), from the top hypermethylated genes were selected and in silico expression analysis based on Oncomine database platform was carried out. Among the 22 genes 9 including KISS1 [26], SERPINB5 [27], and CAMK2B [28] are previously established methylation markers in cancer or other diseases, meanwhile 13 genes including LILRB4, CLDN15 are novel genes of which methylation status has not been known. As shown in Fig. 4 and Fig. S4, all the genes were dysregulated according to the methylation status in tumor cell lines with downregulation in case of hypermethylation and vice versa. To further examine the relation between the expression and methylation, real-time RT-PCR analysis for five genes, one from each cancer, was carried out with RNA isolated from cultured cells composed of a pair of normal and cancer cell line for the five cancers. The result confirmed the fact that all the genes were up or downregulated according to their methylation status in the cancer cell (Fig. 5).

## Discussion

In this study, a key regulatory network was constructed from genes affected by promoter methylation in the highly incident five cancers. In addition, the relationship of the cancer types posed by the genome-wide methylation change was elucidated. So far, cancer classification has been made based on the mRNA expression profiles [29]–[31]. Epigenetic changes have emerged as an alternative explanation to the signatures of cancer development in time courses as well as in different tissues. Recently, a quantitative atlas of histone modification signatures from 24 human cancer cells revealed clusters of HEK293 and U2OS; HFF, HeLa, and PC3; and MCF7, PANC1 and MDAMB231. The data showed a consistency between the mRNA levels and the histone modifying enzymes [32]. In another study, the cancer heterogeneity across cancer types was addressed by the methylation variation in epigenetic domains, with tight clustering of methylation levels in normal tissues, and marked stochastic variation in cancers [33]. These data suggest future efforts might instead be directed at defining the cancer epigenome as the departure from a narrowly defined normal profile.

We found that breast cancer was closest with lung cancer and farthest from colon cancer. Colon cancer is notable in that most methylation alterations occur not in promoters, and also not in CpG islands, but in sequences up to 2 kb distant, the so called CpG island shores [34]. This unique epigenome characteristic might put colon cancer farthest from breast cancer. Breast and lung cancer have many common prognostic signatures, such as hypermethylation of BRCA1 [35], SOX17 [36], [37], and TLX3.

It is interesting that the cluster that was constructed from the 90 cancer-related genes showed a similar pattern at a certain level with that constructed from the whole genes, implying the cancer-related genes undergo a similar methylation change with that of the genome-wide change. It is therefore helpful to chase the key cancer-related genes when addressing the methylome status of a specific cancer.

Notably, each cancer type represented a unique core pathway in the highest confidence network, implying that there appears to be a tissue-specific epigenetic cancer pathway. In the pathway, we found previously known epigenetic markers as well as novel markers that could be developed as epigenetic markers for cancer prognosis. In breast cancer, FLRT2 and SCTR are good candidates for novel markers; the expression of which is known to be down regulated [38], [39]. However, no information for their epigenetic control is yet available.

A limitation of our study is seen in the inability to assess methylation levels with respect to tumor grade, which thus precludes an examination of potential methylation patterns specific to cancer initiation versus those specific to tumor progression. In addition, detecting the frequency of methylation in tumor samples was limited due to the small number of databases for each cancer. Nonetheless, the degree of consistency between our findings and prior literature in the sampling of cancer-relevant genes gives bearing to the potential of using organ-specific alterations in methylation pattern as a prognostic tool in each cancer development and progression.

In summary, we have presented a genome-wide methylation profile of five cancers, colon, liver, lung, gastric, and breast cancer, which we have compiled utilizing databases obtained from the most comprehensive methylation measurement technique currently available. Our findings have provided information about the interrelationship among the five cancers with respect to methylome change. Moreover, we have found a key epigenetic regulatory pathway that is unique in each cancer. Further research into the mechanism leading to the common or differential methylation in the cancers could give insights for estimating prognosis and determining treatment options.

## Supporting Information

Figure S1 Cluster analysis with genes of altered methylation for five cancers. Cluster analysis was carried out using a methylation index (β-value) taken from Illumina Human Methylation 27 Bead Chip database registered at the GEO. A. Hierarchical clustering with the correlation distance was shown for 10 tumor tissues and 10 normal tissues from each of the breast, colon, liver, lung, and stomach cancer. B. Significance analysis of microarray (SAM) for the 10 colon cancer tissues. Genes showing differential methylation among the 10 tissues were represented by the heatmap analysis. The color bar at top denotes the methylation level.
(DOCX)

Figure S2 Highest confidence network of genes displaying altered methylation levels in colon, liver, lung, and stomach cancer. In the network, hypermethylated genes in cancer are colored in red, whereas the hypomethylated genes are shown in green. The intensity of the color reflects the magnitude of methylation change. According to IPA, the network is relevant to: cellular growth and proliferation, cancer, connective tissue disorders in colon cancer (A); post-translational modification, protein degradation, protein synthesis in liver cancer (B); cancer, dermatological diseases and conditions, cell death and survival in lung cancer (C); hereditary disorder, neurological disease, cellular assembly and organization in stomach cancer (D).
(DOCX)

Figure S3 Top functional pathways most strongly associated with the significantly altered genes in colon, liver, lung, and stomach cancer. Top functional categories are given for colon (A), liver (B), lung (C), and stomach cancer (D). The Ingenuity software assigns a *P* value based on the likelihood of obtaining the observed number of category or pathway-related molecules in a given data set by chance alone. The threshold line denotes the *p* = 0.05 level. The line graph represents the ratio of affected genes to the total number of genes in a pathway.
(DOCX)

Figure S4 In silico expression profiles of selected genes with altered methylation in colon, liver, lung, and stomach cancer. Five genes (two in case of liver) that have shown methylation change in each cancer were selected, and their expression was examined in silico in the expression dataset and represented as a box plot; colon (A), liver (B), lung (C), and stomach cancer (D). There appeared a coincidence that hypermethylated genes showed downregulation, or that hypomethylated genes showed upregulation in cancer.
(DOCX)

Table S1 Genome-wide methylation databases analyzed in this study.
(DOCX)

Table S2 Sequences of primers employed in this study.
(DOCX)
