# Supplementary figures and images for: Identification and Comparison of Aberrant Key Regulatory Networks in Breast, Colon, Liver, Lung, and Stomach Cancers through Methylome Database Analysis
Source: PLoS One. 2014 May 19;9(5):e97818. doi: 10.1371/journal.pone.0097818 (PMC4026530; doi:10.1371/journal.pone.0097818)

A


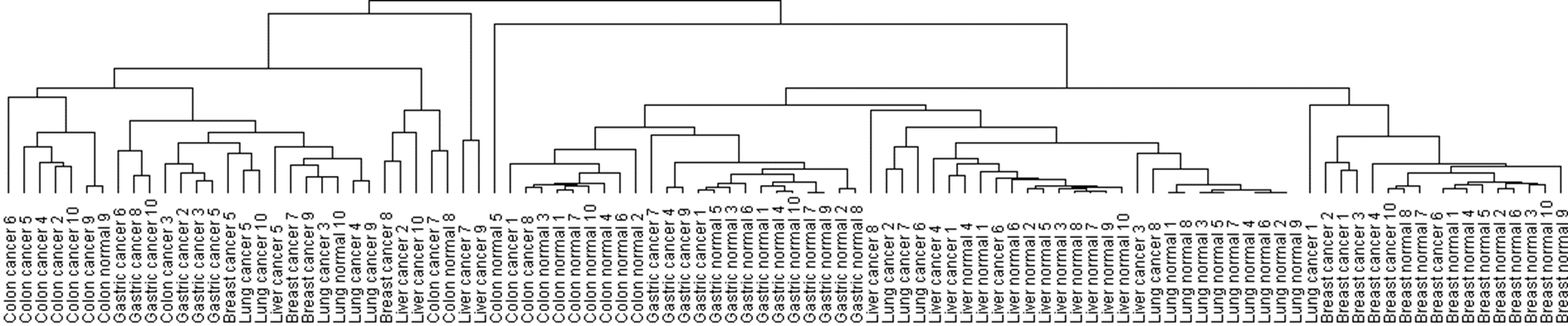


B


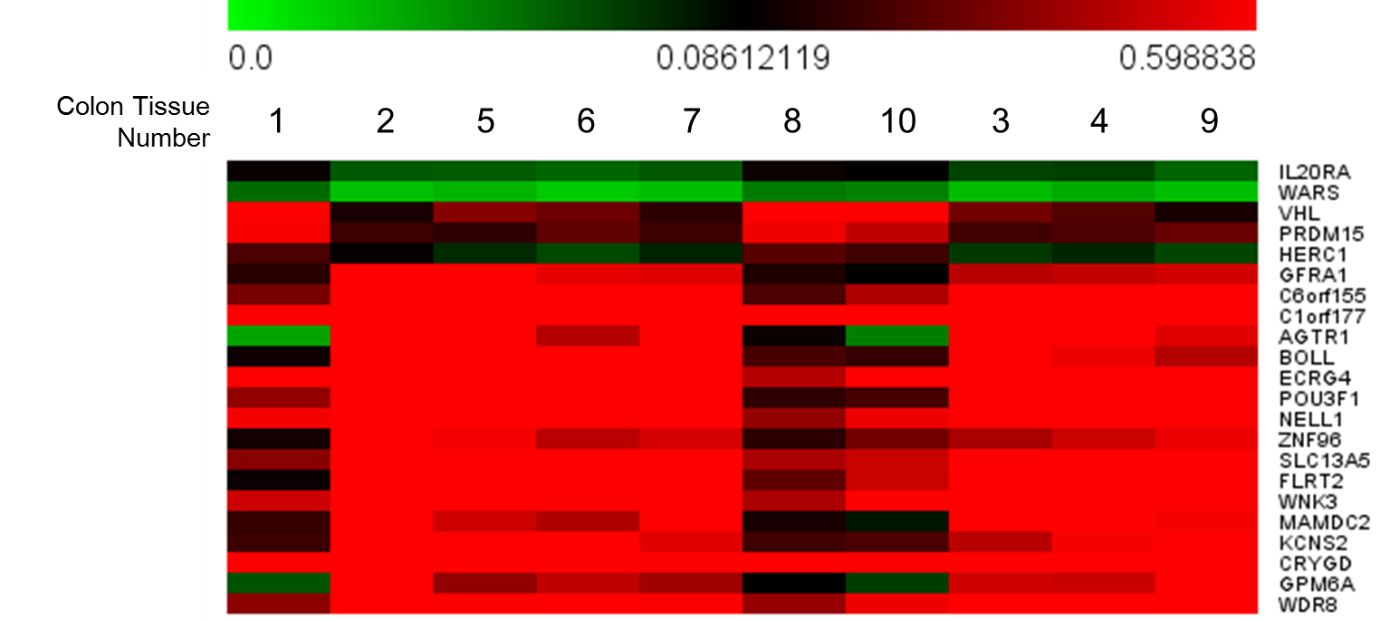

Supplement: Figure S1 — Cluster analysis with genes of altered methylation for five cancers. Cluster analysis was carried out using a methylation index (β-value) taken from Illumina Human Methylation 27 Bead Chip database registered at the GEO. A. Hierarchical clustering with the correlation distance was shown for 10 tumor tissues and 10 normal tissues from each of the breast, colon, liver, lung, and stomach cancer. B. Significance analysis of microarray (SAM) for the 10 colon cancer tissues. Genes showing differential methylation among the 10 tissues were represented by the heatmap analysis. The color bar at top denotes the methylation level. (DOCX) [file pone.0097818.s001.docx]

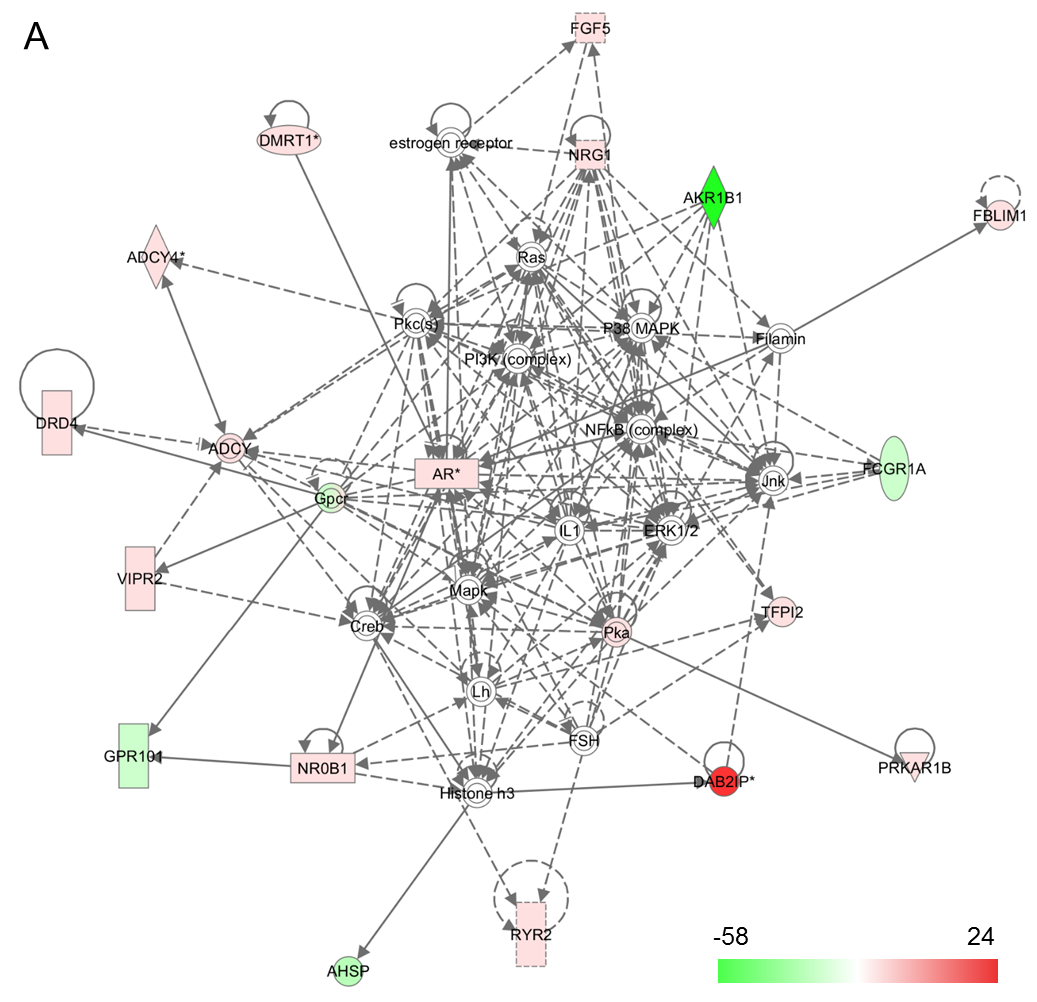


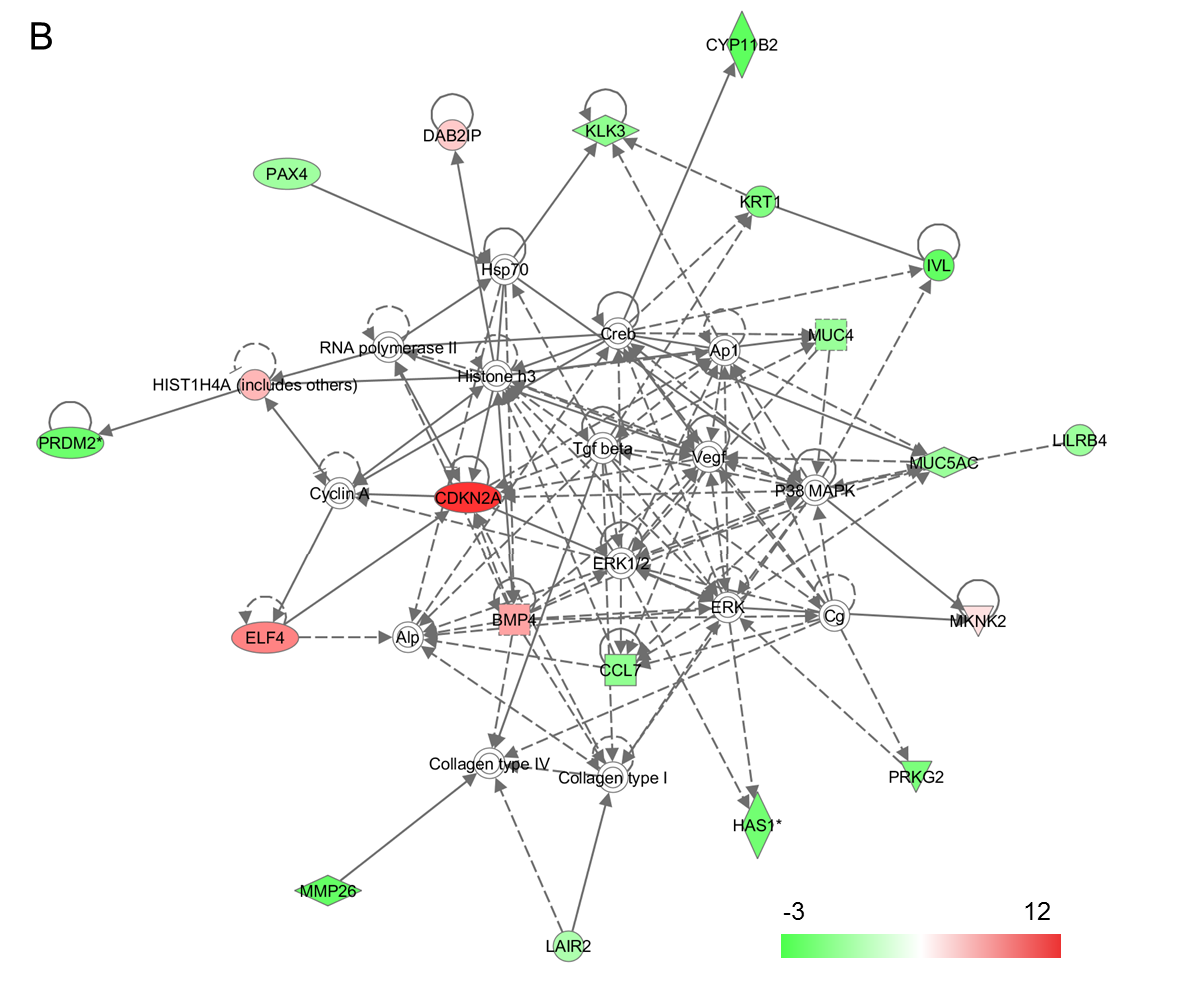


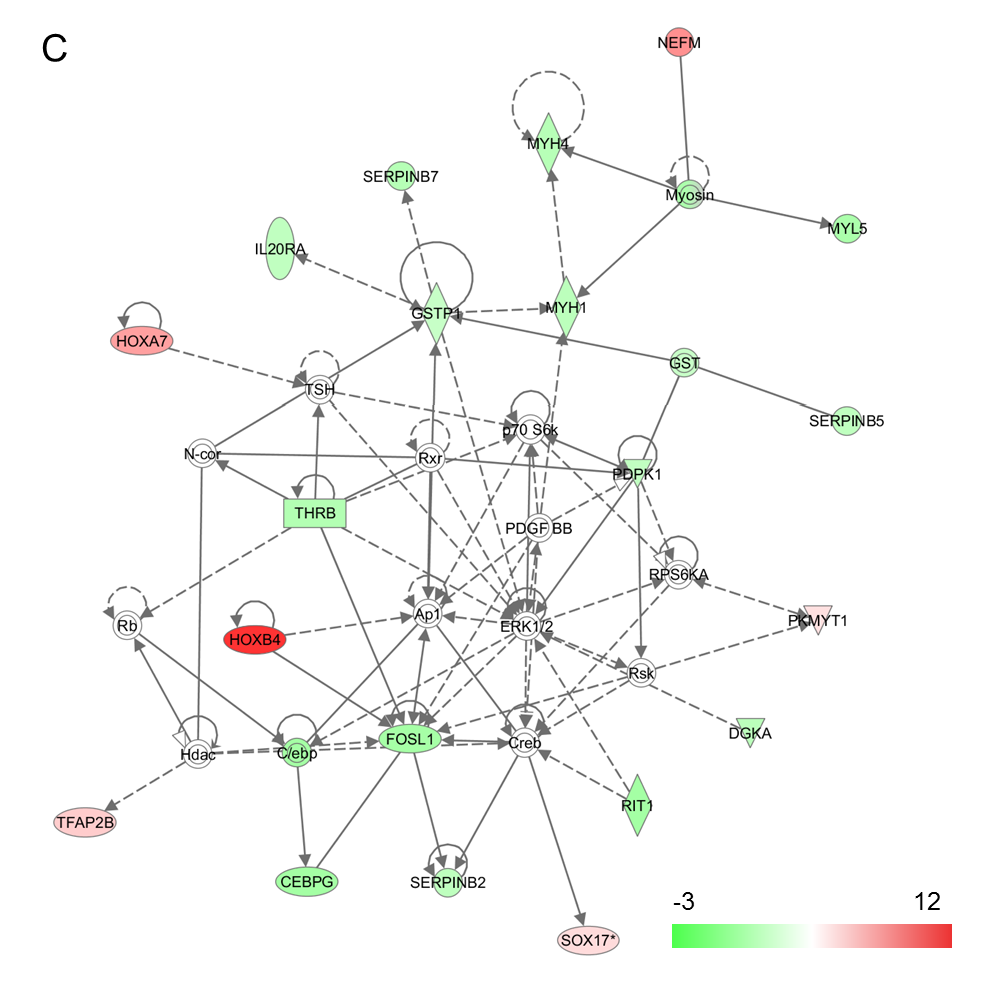


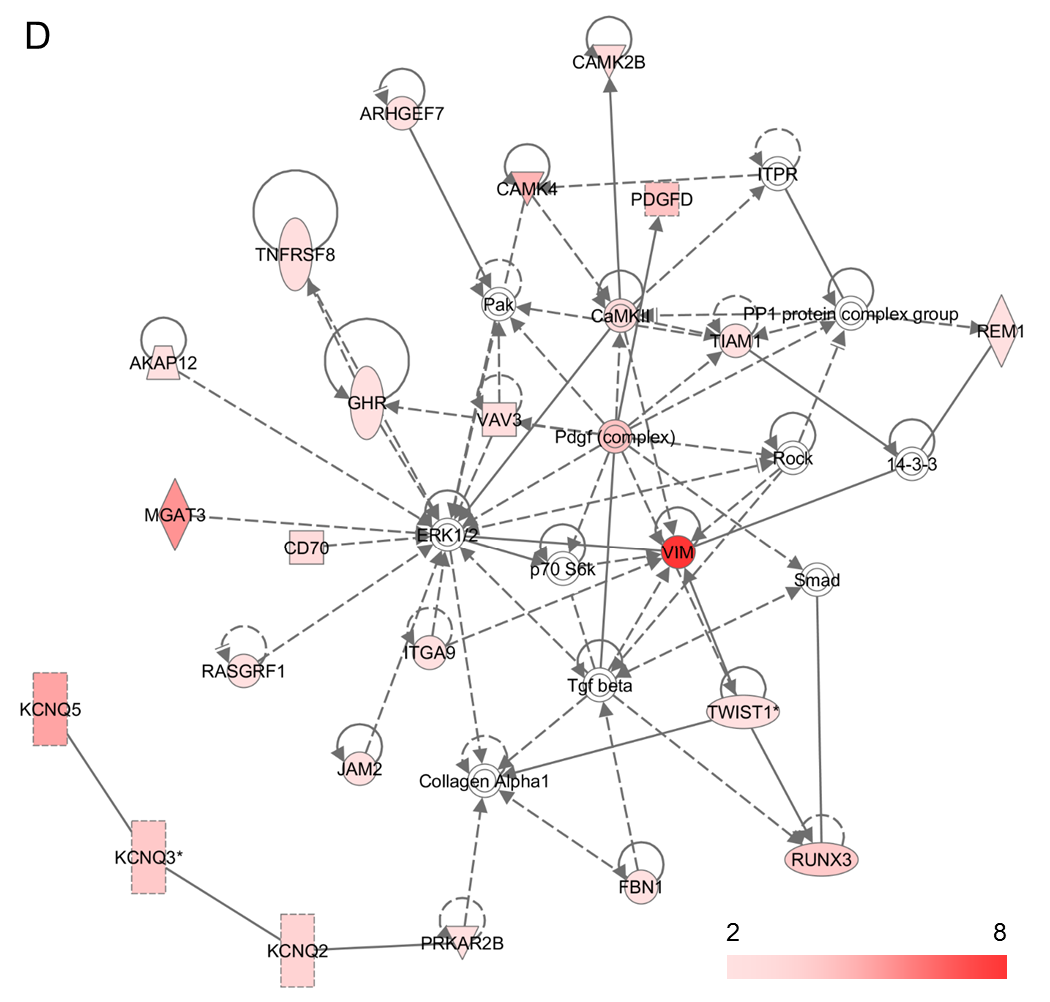

Supplement: Figure S2 — Highest confidence network of genes displaying altered methylation levels in colon, liver, lung, and stomach cancer. In the network, hypermethylated genes in cancer are colored in red, whereas the hypomethylated genes are shown in green. The intensity of the color reflects the magnitude of methylation change. According to IPA, the network is relevant to: cellular growth and proliferation, cancer, connective tissue disorders in colon cancer (A); post-translational modification, protein degradation, protein synthesis in liver cancer (B); cancer, dermatological diseases and conditions, cell death and survival in lung cancer (C); hereditary disorder, neurological disease, cellular assembly and organization in stomach cancer (D). (DOCX) [file pone.0097818.s002.docx]

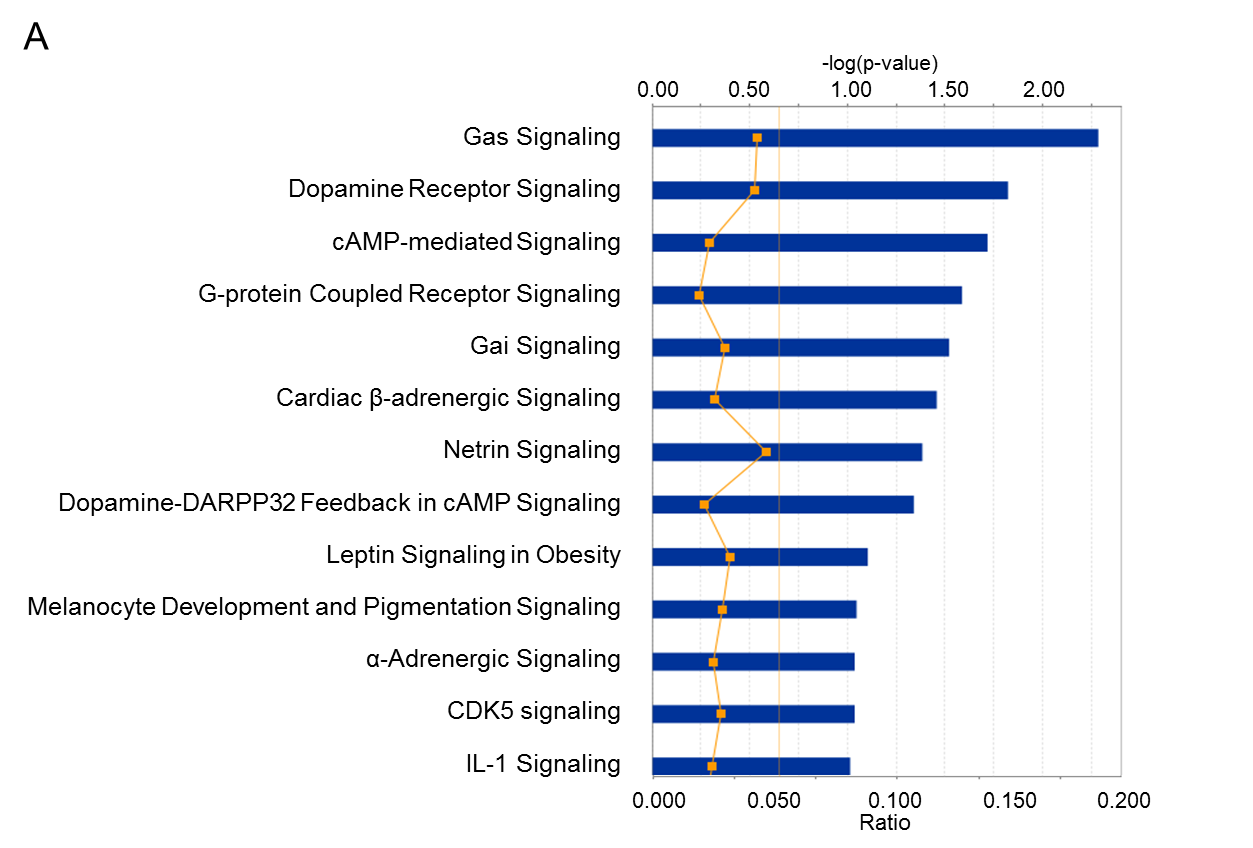


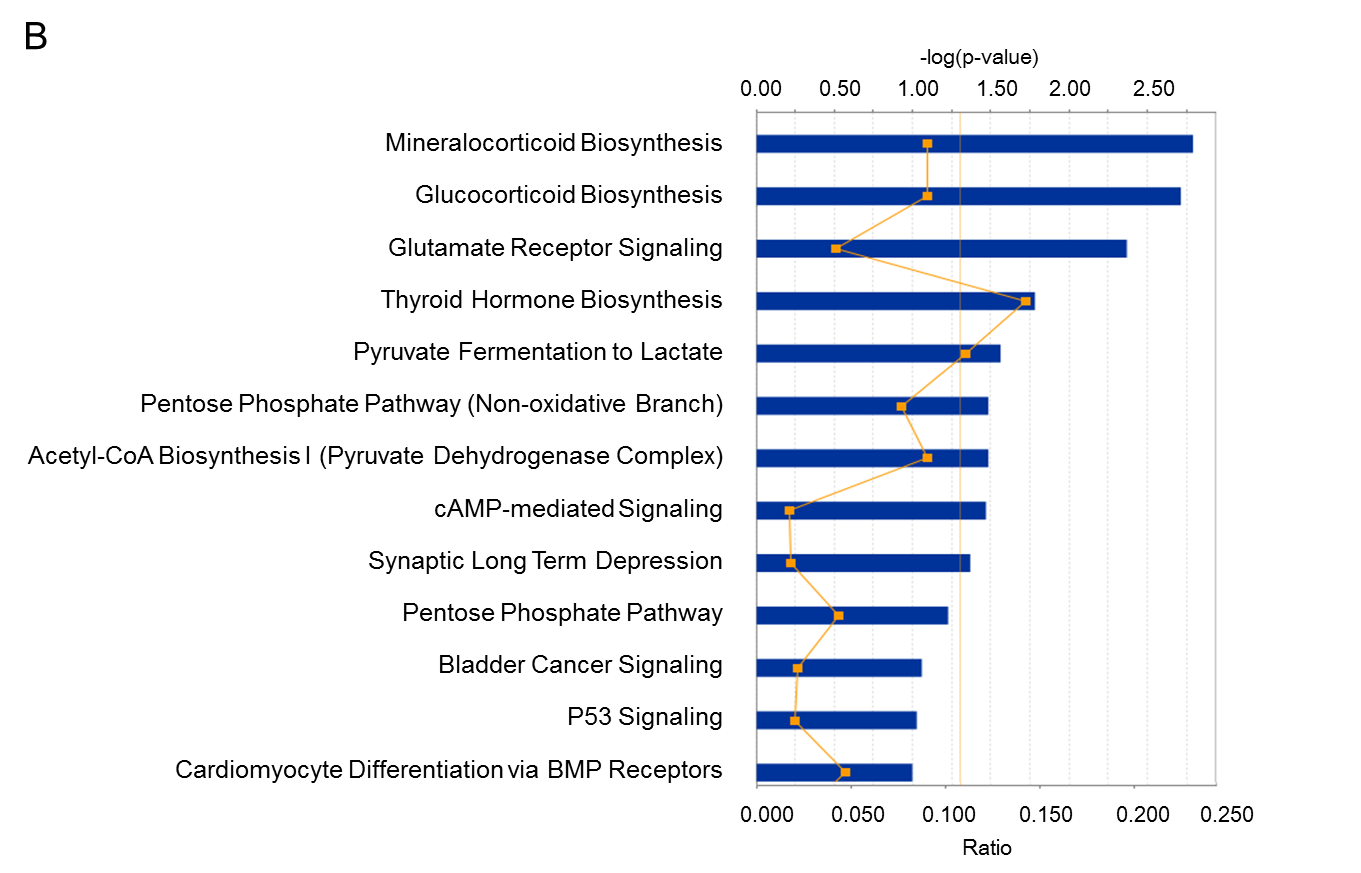


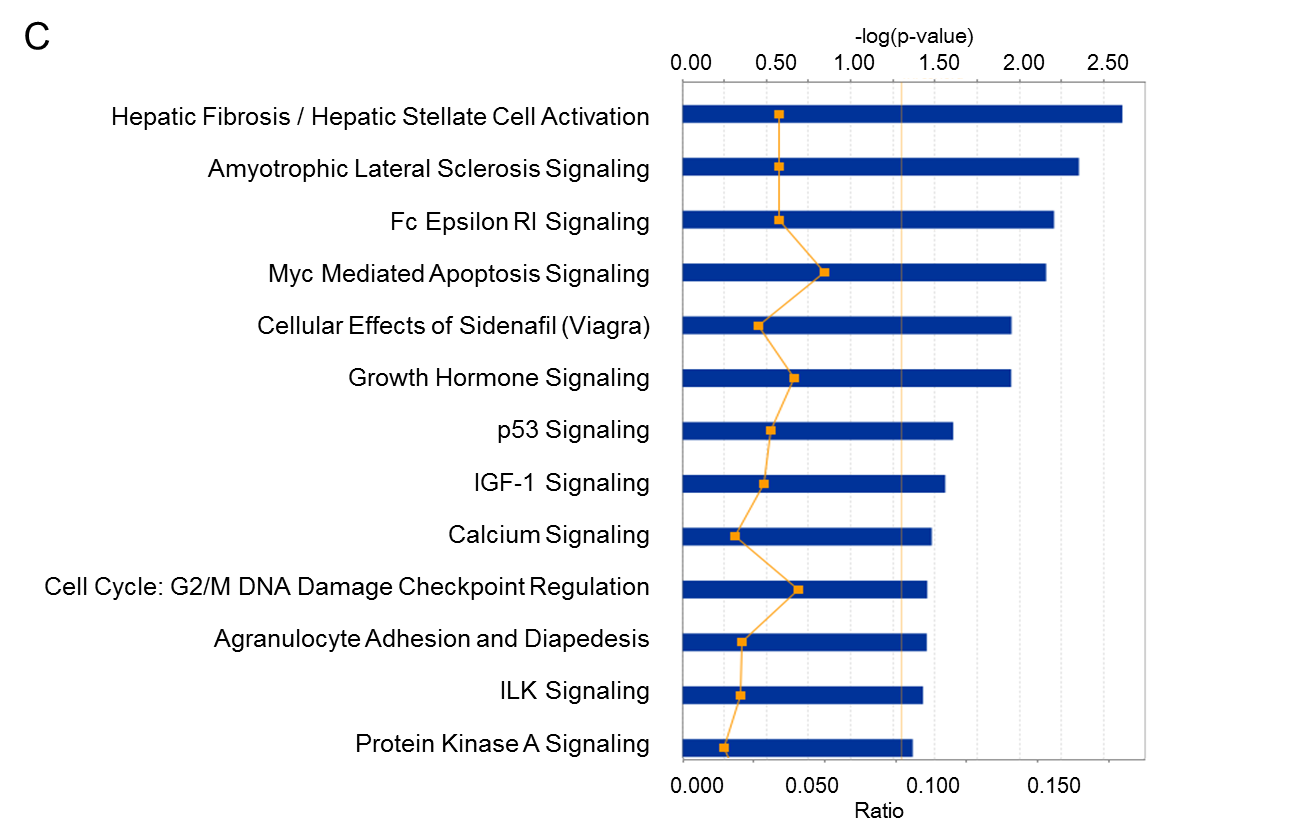


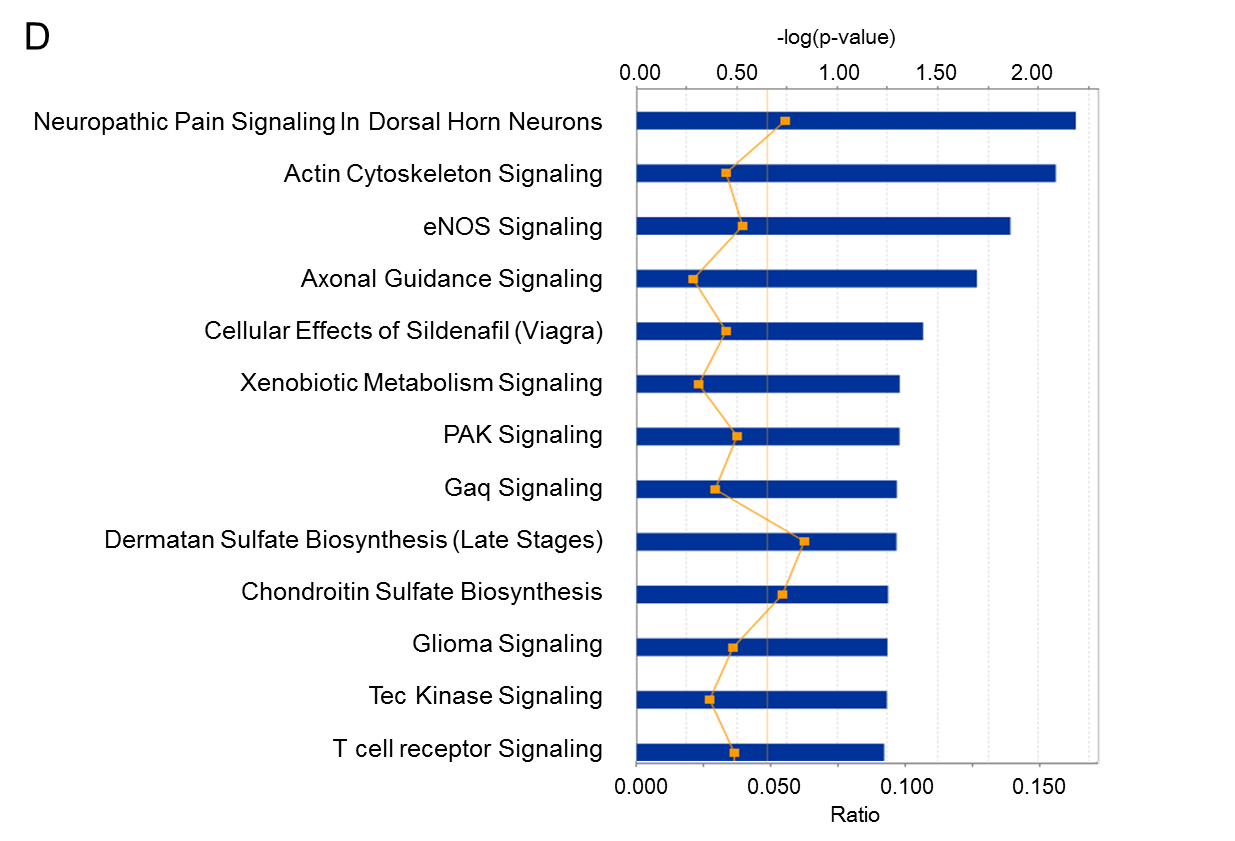

Supplement: Figure S3 — Top functional pathways most strongly associated with the significantly altered genes in colon, liver, lung, and stomach cancer. Top functional categories are given for colon (A), liver (B), lung (C), and stomach cancer (D). The Ingenuity software assigns a P value based on the likelihood of obtaining the observed number of category or pathway-related molecules in a given data set by chance alone. The threshold line denotes the p = 0.05 level. The line graph represents the ratio of affected genes to the total number of genes in a pathway. (DOCX) [file pone.0097818.s003.docx]

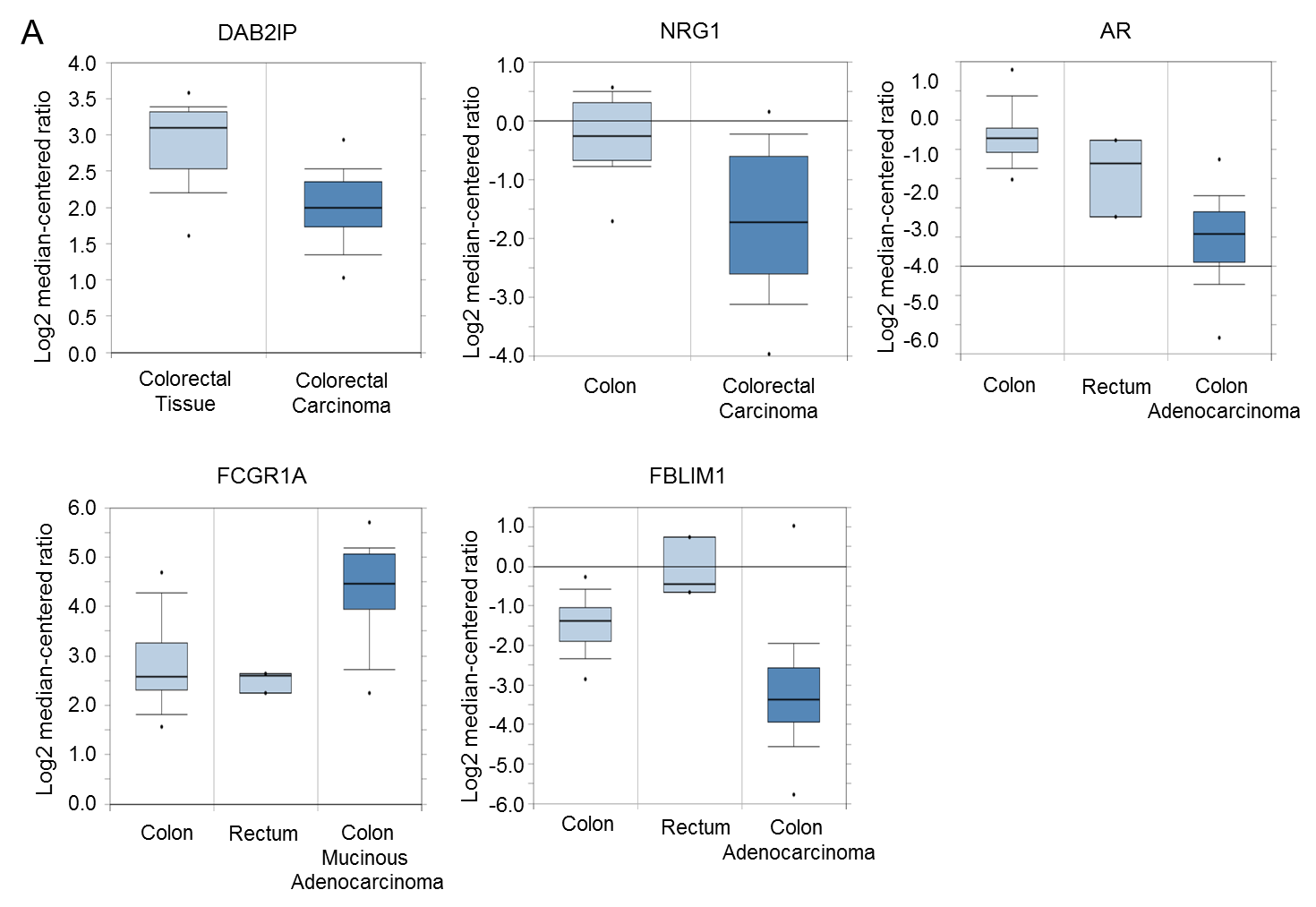


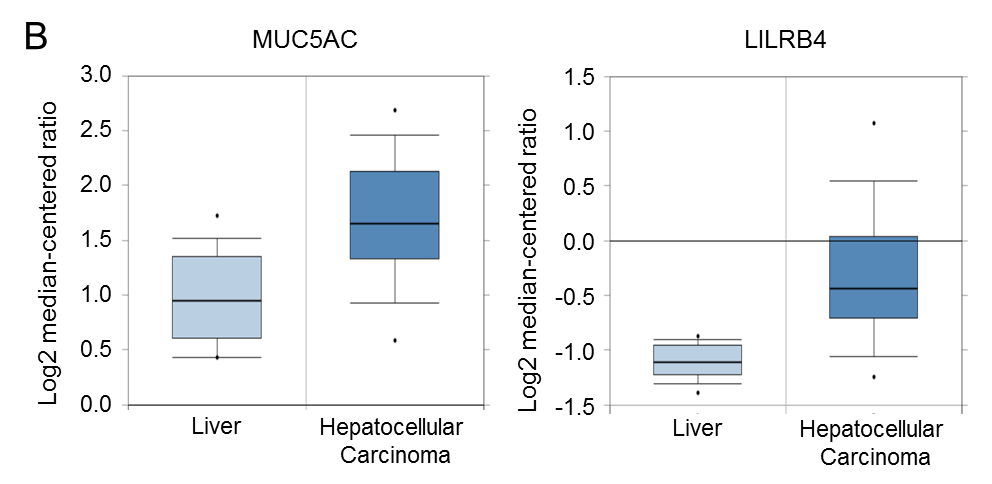


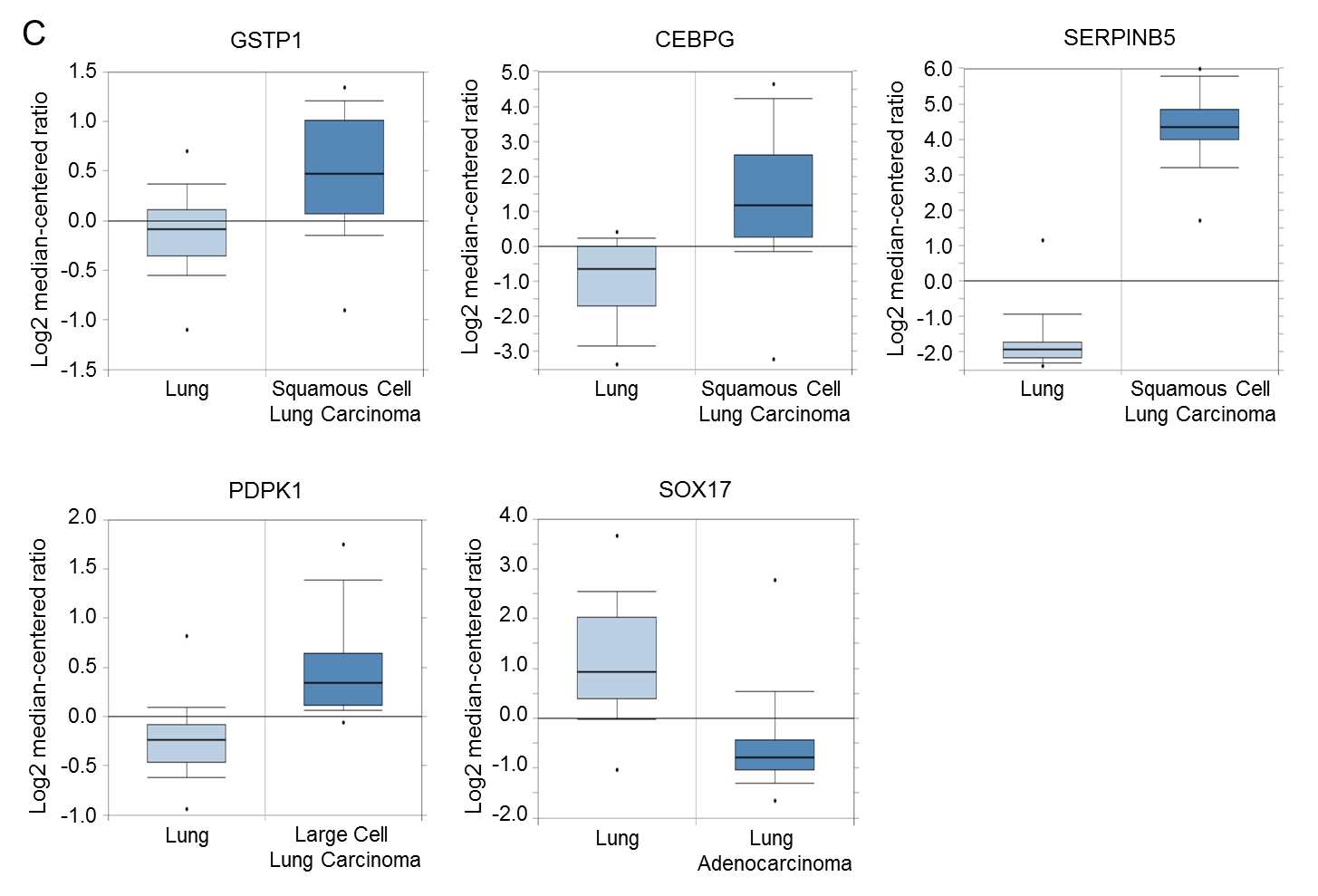


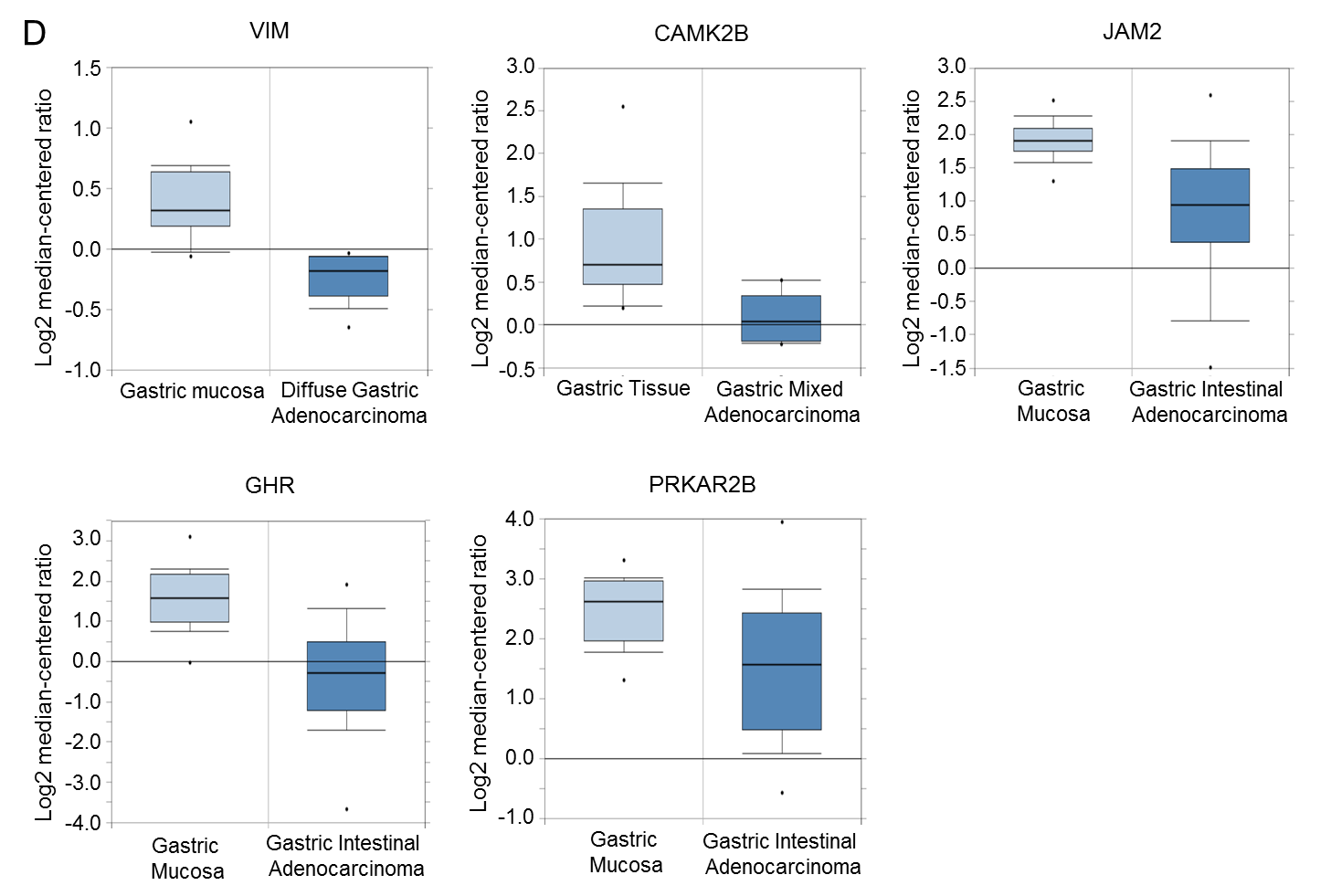

Supplement: Figure S4 — In silico expression profiles of selected genes with altered methylation in colon, liver, lung, and stomach cancer. Five genes (two in case of liver) that have shown methylation change in each cancer were selected, and their expression was examined in silico in the expression dataset and represented as a box plot; colon (A), liver (B), lung (C), and stomach cancer (D). There appeared a coincidence that hypermethylated genes showed downregulation, or that hypomethylated genes showed upregulation in cancer. (DOCX) [file pone.0097818.s004.docx]
