# Supplementary material for: Identification and Comparison of Aberrant Key Regulatory Networks in Breast, Colon, Liver, Lung, and Stomach Cancers through Methylome Database Analysis
Source: PLoS One. 2014 May 19;9(5):e97818. doi: 10.1371/journal.pone.0097818 (PMC4026530; doi:10.1371/journal.pone.0097818)
Supplement: Table S1 — Genome-wide methylation databases analyzed in this study. (DOCX) [file pone.0097818.s005.docx]

| **Table S1. Genome-wide methylation databases analyzed in this study.** | | |
| --- | --- | --- |
| Cancer type | | database ID |
| Breast | GSM801718, GSM801706, GSM801707, GSM801708,GSM801709, GSM801710, GSM801711, GSM801712, GSM801713, GSM801714, GSM801715 | |
| Colon | GSM670203,GSM670204, GSM670209, GSM670210, GSM670219, GSM670220, GSM670221, GSM670222, GSM670223, GSM670224, GSM670225, GSM670226, GSM670227, GSM670228, GSM670229, GSM670230, GSM670231, GSM670232, GSM670233, GSM670234 | |
| Liver | GSM931425, GSM931426, GSM931427, GSM931428,GSM931429, GSM931430, GSM931431, GSM931432, GSM931433, GSM931434, GSM931363, GSM931364, GSM931365, GSM931366, GSM931367, GSM931368, GSM931369, GSM931370, GSM931371, GSM931372 | |
| Lung | GSM813174, GSM813175, GSM813180, GSM813181, GSM813184, GSM813185, GSM813208, GSM813209, GSM813210, GSM813211, GSM813212, GSM813213, GSM813214, GSM813215, GSM813232, GSM813233, GSM813234, GSM813235, GSM813236, GSM813237 | |
| Stomach | GSM635137, GSM635138, GSM635139, GSM635140, GSM635141, GSM635142, GSM635143, GSM635144, GSM635145, GSM635146,  GSM635147, GSM635148, GSM635149, GSM635150, GSM635151, GSM635152, GSM635153, GSM635154, GSM635155, GSM635156 | |
