# Supplementary material for: Identification and Comparison of Aberrant Key Regulatory Networks in Breast, Colon, Liver, Lung, and Stomach Cancers through Methylome Database Analysis
Source: PLoS One. 2014 May 19;9(5):e97818. doi: 10.1371/journal.pone.0097818 (PMC4026530; doi:10.1371/journal.pone.0097818)
Supplement: Table S2 — Sequences of primers employed in this study. (DOCX) [file pone.0097818.s006.docx]

| **Table S2. Sequences of primers employed in this study.** | |  |
| --- | --- | --- |
| **Genes** | **Forward primer (5’–3’)** | **Reverse primer (5’–3’)** |
| **Real-time RT-PCR** |  |  |
| GAPDH | CAGGAGGCATTGCTGATGAT | GAAGGCTGGGGCTCATTT |
| KISS1 | CCTCTGGACATTCACCCAGC | CCTAGAAGTGCCTTGAGGCTTG |
| FCGR1A | GCAAGTGGACACCACAAAGG | AGTGGCTGTGCCATTGAGAA |
| SOX17 | AAGATGCTGGGCAAGTCGTG | GCGGCCGGTACTTGTAGTT |
| VIM | ACGTCTTGACCTTGAACGCA | TCCTGGATTTCCTCTTCGTGG |
| LILRB4 | AAAACTTCTGTGCTGCCGTG | TGGAGTGTTTCACCTTGGCA |
